# Chelation of Cu(II), Zn(II), and Fe(II) by Tannin Constituents of Selected Edible Nuts

**DOI:** 10.3390/ijms10125485

**Published:** 2009-12-22

**Authors:** Magdalena Karamać

**Affiliations:** Division of Food Science, Institute of Animal Reproduction and Food Research, Polish Academy of Sciences, Tuwima 10, 10-747 Olsztyn, Poland; E-Mail: m.karamac@pan.olsztyn.pl; Tel.: +48-895-234-627; Fax: +48-895-240-124

**Keywords:** walnuts, hazelnuts, almonds, tannins, metal chelating capacity, Fe(II), Cu(II), Zn(II)

## Abstract

The tannin fractions isolated from hazelnuts, walnuts and almonds were characterised by colorimetric assays and by an SE-HPLC technique. The complexation of Cu(II) and Zn(II) was determined by the reaction with tetramethylmurexide, whereas for Fe(II), ferrozine was employed. The walnut tannins exhibited a significantly weaker reaction with the vanillin/HCl reagent than hazelnut and almond tannins, but the protein precipitation capacity of the walnut fraction was high. The SE-HPLC chromatogram of the tannin fraction from hazelnuts revealed the presence of oligomers with higher molecular weights compared to that of almonds. Copper ions were most effectively chelated by the constituents of the tannin fractions of hazelnuts, walnuts and almonds. At a 0.2 mg/assay addition level, the walnut tannins complexed almost 100% Cu(II). The Fe(II) complexation capacities of the tannin fractions of walnuts and hazelnuts were weaker in comparison to that of the almond tannin fraction, which at a 2.5 mg/assay addition level, bound Fe(II) by ~90%. The capacity to chelate Zn(II) was quite varied for the different nut tannin fractions: almond tannins bound as much as 84% Zn(II), whereas the value for walnut tannins was only 8.7%; and for hazelnut tannins, no Zn(II) chelation took place at the levels tested.

## 1. Introduction

Phenolic compounds constitute a very diversified group of plant secondary metabolites in terms of structure, molecular weight and physicochemical and biological properties. Edible nuts, among others, can serve as a source of phenolic compounds in a human diet [1]. Phenolic compounds of nuts are located mainly in the skins covering kernels. Removing of skin from walnuts decreased the total phenolic content by 90%, approximately, and in case of other nuts by *ca*. 50% [2]. Almonds, hazelnuts and walnuts contain a variety of low-molecular-weight phenolic compounds, including phenolic acids (caffeic, *p*-coumaric, protocatechuic, vanillic, gallic, sinapic, *p*-hydroxybenzoic, chlorogenic, ellagic acids), flavonols (quercetin, isorhamnetin, kaempferol, morin) and/or their glycosides, flavanones (naringenin, eriodictyol) and/or their glycosides, flavan-3-ol monomers (catechin, epicatechin), dimers and trimers. [3–7]. The above mentioned nuts are also rich in tannins. Condensed tannins with degrees of polymerisation up to 10 (oligomers), as well as higher (polymers) are present in seeds of all those nuts [8]. Tannin fractions comprising constituents reacting with vanillin/HCl reagent, were isolated using Sephadex LH-20 column chromatography from almond kernels [9] as well as skins of hazelnuts [10]. The richest source of condensed tannins among the above mentioned nuts is hazelnuts and they comprise (epi)catechin or (epi)gallocatechin subunits [8]. All three flavan-3-ols, which are common in proanthocyanidin structures: (epi)catechin, (epi)gallocatechin and (epi)afzelechin, were identified as subunits of condensed tannins from almonds [11]. Condensed tannins of walnuts are constituted by (epi)catechin units, however their content in seeds is seven times lower than in hazelnuts [8]. Walnut seeds are characterised by a low content of proanthocyanidins, but a high content of hydrolysable tannins [12]. Fukuda *et al.* [13] and Ito *et al.* [14] identified and isolated more than 20 hydrolysable tannins from the extract of walnuts. Those tannins comprised mainly of ellagitannins, but gallotannins were also present.

Tannins exhibit strong antioxidant properties in comparison to low molecular weight phenolic compounds [9,15,16]. Antioxidant properties of tannins can result from their free radical scavenging activity [15,17,18] but their ability to chelate transition metal ions, especially Fe(II) and Cu(II), also plays an important role. Metal ions can generate highly reactive oxygen free radicals by Fenton or Haber-Weiss chemistry. In the Fenton reaction the hydroxyl radical (HO^•^) is produced from hydrogen peroxide. In the iron-catalysed Haber-Weiss reaction the superoxide radical (O_2_^•−^) reduces ferric to ferrous ions, which then are again involved in generating of hydroxyl radical [19]. Extremely reactive hydroxyl radicals can interact with many biological macro- and small molecules and therefore lead to lipid peroxidation, DNA damage and polymerisation or denaturation of proteins. The binding of transition metal ions by tannins can stabilize prooxidative activity of those ions [20,21].

Literature resources provide papers on metal ion chelating ability of commercially available tannins [20–23]. Yet data on complexing of ion metals by tannins isolated from plant extracts are scarce. Therefore, the aim of this study was to characterise the tannin fractions obtained from three edible nuts and to examine their ability to chelate Fe(II), Cu(II) and Zn(II). The knowledge of those properties can be applied in future studies aimed at elucidating the mechanisms of antioxidant action of tannins present in nut seeds.

### 2. Results and Discussion

Tannin fractions of almonds, walnuts and hazelnuts kernels, obtained using Sephadex LH-20 column chromatography, were characterised by colour reaction with vanillin/HCl reagent enabling determination of condensed tannins content and by the BSA precipitation method allowing evaluation of the protein precipitation capacity of tannins. The results of these assays are shown in Table 1. The content of condensed tannins was 1,261 and 776 mg CE/g for the hazelnut and almond tannin fractions, respectively, whereas the result of this assay for fraction of walnut was several times lower. On the other hand, protein precipitation capacities of hazelnut and walnut tannin fractions were comparable: 940 and 873 mg TAE/g, but a more than tenfold lower value was noted for the almond tannin fraction. The content of condensed tannins in the fraction isolated from hazelnut kernels was more than twice as low as the value reported by Alasalvar *et al.* [10] for a fraction isolated from hazelnut skins. Gu *et al.* [24] determined proanthocyanidins present in nuts seeds, among others, using HPLC-MS/MS analysis. They noted that total proanthocyanidin contents for nuts included in the present study decreased in the order hazelnuts > almonds ≫ walnuts.

The obtained results indicated that tannin fraction from walnuts was characterised by its low proanthocyanidin content, but simultaneously suggested that it contained hydrolysable tannins, which do not react with vanillin/HCl reagent but are able to precipitate BSA. The presence of hydrolysable tannins (mainly ellagitannins) in walnuts was reported by Fukuda *et al.* [13] and Ito *et al.* [14]. High molecular weight hydrolysable tannins are insoluble and/or covalently bound to the cell wall. However, Li *et al*. [6] reported, that more than half of ellagitannins (determined as ellagic acid liberated after acidic hydrolysis) present in walnuts seeds was not bound and are thus extractable with 80% (v/v) methanol.

**Figure 1.**
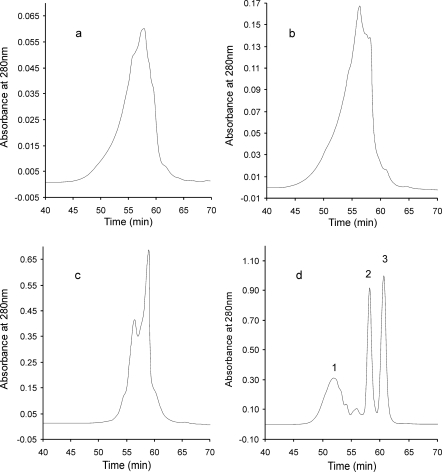
SE-HPLC chromatograms of tannin fractions of almonds (a), hazelnuts (b) and walnuts (c) as well as standards: 1 - tannic acid, 2 - procyanidin B_2_, 3 - gallic acid (d).

**Figure 2.**
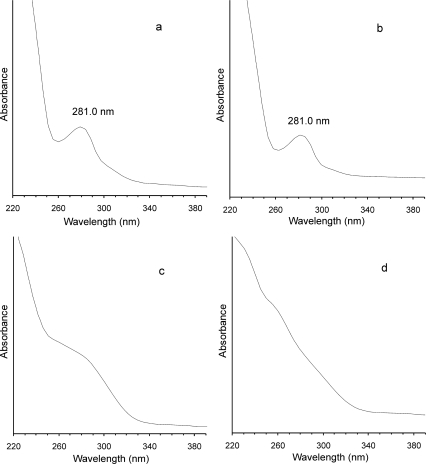
UV-spectra of peaks from SE-HPLC chromatograms (Figure 1): (a) - of peak with retention time (t_r_) 57.0 min of almond tannins separation, (b) - of peak with t_r_ 55.1 min of hazelnut tannins separation, (c) - of peak with t_r_ 55.8 min and (d) - t_r_ 58.0 min of walnut tannins separations.

The tannin fraction from almonds comprised constituents which react with vanillin/HCl reagent, but precipitate BSA to a small extent, since they are probably mainly condensed tannins with low degrees of polymerisation. Dimers of condensed tannins are less effective precipitating agents [25]. Also oligomers containing three subunits are not always precipitated by proteins [26]. The low degree of polymerisation of the proanthocyanidins of the almond fractions was confirmed by SE-HPLC analysis (Figure 1a). In the chromatogram of this fraction a peak with a retention time (t_r_) of about 57 min was predominant. This peak was related to the molecular weight of dimer-procyanidin B_2_ and higher (Figure 1d). Oligomers of molecular weights larger than that of procyanidin B_2_, but smaller than tannic acid were present also in almond fraction. On the other hand, in the chromatogram of the hazelnut fraction (Figure 1b) a highest peak with shorter t_r_ of 55 min was noted. The UV spectra of the discussed compounds are similar for both analysed fractions, with maxima at 281 nm (Figure 2a and 2b).

In the SE-HPLC chromatogram of the walnut tannin fraction two separated peaks are present (Figure 1c). The peak with retention time of 58 min, corresponding to z molecular weight of about 600 is predominant. The peak with shorter t_r_ of 56 min originated from constituents of molecular weight larger than procyanidin B_2_, but smaller than that of tannic acid. UV spectra recorded at those retention times did not reveal any maxima and were characterised by shoulders only (Figure 2c and 2d). Then, hydrolysable tannins, which are predominant in the tannin fraction of walnuts, are molecules of molecular weights corresponding to strictinin (ellagitanin) or trigalloyl derivatives (gallotannin) and larger. To our knowledge, there is no literature data about the precipitating ability of low molecular weight ellagitannins with proteins. In case of gallotannins the smallest esters of glucose able to precipitate with BSA are molecules with at least three galloyl groups [27]. It seems that the smallest gallotannin able to precipitate BSA has a molecular weight of 636.5 (β-1,3,6-tri-*O*-galloyl-d-glucose). On the other hand, the condensed tannins molecules with molecular weight about 600 do not precipitate proteins [25]. This provides an explanation for the high protein precipitation capacity of the tannin fraction from walnuts, which differentiates them from almond tannins, despite the comparatively low molecular weights of the constituents of both fractions.

Chromatograms of SE-HPLC revealed that all the studied nut tannin fractions were characterised by the presence of constituents with lower molecular weights than the tannin fractions from buckwheat seeds and groats isolated and separated under analogous conditions [28].

**Figure 3.**
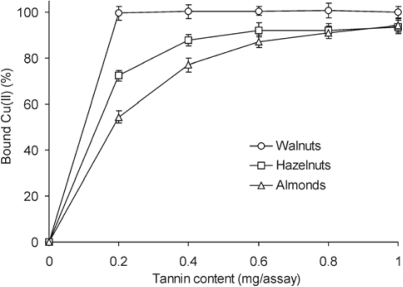
Cu(II) chelation capacity of nuts tannin fractions. Results are expressed as means ± standard deviations (*n* = 3).

The Cu(II) and Zn(II) chelating capacity of tannins from the examined nuts was determined by the assay with tetramethylmurexide (TMM). “Free” metal ions, which were not bound by tannins, were complexed with TMM. TMM solution showed absorption maximum at 530 nm, and complex formed of TMM with Cu(II) or Zn(II) at 482 nm and 462 nm, respectively. The ratio of absorbance measured at both wavelengths allows one to estimate the amount of Cu(II) or Zn(II) complexed with TMM. Knowing the total quantity of metal ions added to the reaction mixture, the % of Cu(II) or Zn(II) bound by the test tannin fraction can be calculated by difference. Copper ions were effectively chelated by the tannin constituents of all three analysed samples (Figure 3). At a 0.2 mg/assay addition level, the walnut tannins complexed almost 100% Cu(II). The same quantity of hazelnut and almond tannins bound only 72.3% and 54.5% Cu(II), respectively. Above 90% Cu(II) was chelated by hazelnut and almond tannins at the addition levels of 0.6 and 0.8 mg/assay, respectively. The greater addition of tannins tested (1 mg/assay) bound 93.5% and 94.3% copper ions. The same level of chelating of copper ions was noted for tannin fractions of buckwheat seeds [28]. Wong *et al.* [29] tested Cu(II) chelating ability of extracts of 25 edible tropical plants and established that they complexed from 40 to 95% of copper ions added.

**Figure 4.**
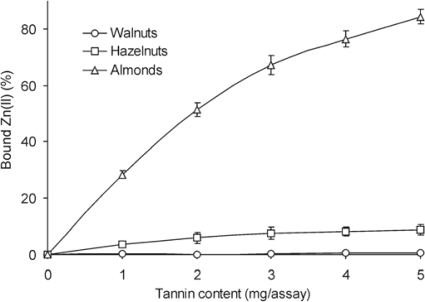
Zn(II) chelation capacity of nuts tannins fractions. Results are expressed as means ± standard deviations (*n* = 3).

The capacity to chelate Zn(II) was quite varied for the different nut tannins (Figure 4). Tannin fraction from almonds bound zinc ions much more effectively than the other two. At the lowest addition level applied - 1 mg/assay, the % of Zn(II) chelated amounted to 28.4%, and at a 5 mg/assay addition level tannins bound as much as 84% Zn(II). However, the value was only 8.7% for walnut tannins at the highest level of sample addition. Hazelnut tannins virtually did not complex Zn(II) ions; the greatest addition of ligand bound less than 1% of zinc ions. For comparison, buckwheat seeds and groats tannins complexed Zn(II) ions much weaker than the analysed fraction from almonds, but several percent stronger than walnut tannins [28], whereas 0.1% solution of instant coffee chelated 100% Zn(II) at pH 6.0 and about 75% at pH 5.0 [30].

**Figure 5.**
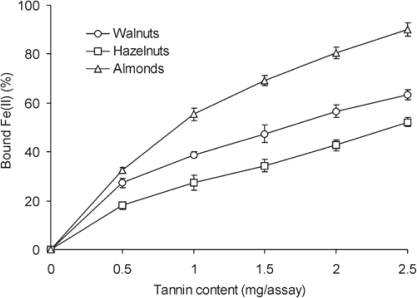
Fe(II) chelation capacity of nuts tannin fraction. Results are expressed as means ± standard deviations (*n* = 3).

The TMM method should not be used to evaluate the iron ion chelation capacity of tannins and plant extracts rich in tannin constituents [31]. Therefore, the method with ferrozine was employed for examination of Fe(II) chelating capacity of the tannin fractions of the tested nuts. Ferrozine forms with bivalent iron ions stable, colourful complexes with a high extinction coefficient at 562 nm. Figure 5 depicts the Fe(II) chelation capacity of the tannin fractions of almonds, hazelnuts and walnuts. The addition of 0.5 to 2.5 mg of tannin fraction per assay caused the increase of the amount of bound Fe(II). The largest increase was noted for almond tannins, then walnuts tannins and the least for hazelnuts. A 2.5 mg sample of tannins gave complexation of 90.1, 63.4 and 52.2% of the total quantity of Fe(II) added to the reaction mixture, respectively. The good Fe (II) chelating properties of almond tannins confirmed the results obtained by Wijeratne *et al.* [4]. They revealed that 200 ppm of extract from whole seed and brown skin of almonds complexed 97% and 98%, respectively from 400 μM of ferrous ions. On the other hand buckwheat seeds and groats tannins, obtained in the same manner as in the present study, chelated slightly less Fe(II), 53% and 24%, respectively [28].

In order to compare the ability of tannins fractions to chelate copper, iron and zinc ions, the amount of μmol of Fe(II), Cu(II) and Zn(II) was complexed by a constant amount (0.5 mg) of nut fraction tested was estimated (Table 2). Tannin fractions from nuts chelated copper ions the most effectively. The highest amount of Cu(II) was chelated by tannins from walnuts (25.0 μmol), and the lowest by tannins from almonds (20.5 μmol). Iron ions were complexed much weaker than copper ions. The ability of tannin fractions to chelate iron ions was in the range from 1.8 to 3.2 μmol Fe(II). Zn (II) was bound by tannins from walnuts and hazelnuts the least effectively. Only the almonds fraction chelated more zinc than iron ions. The chelating effectiveness of tested tannin fractions decreased in the following order: Cu(II) > Fe(II) > Zn(II), what was in line with other researchers’ results. Mira *et al.* [32] compared the degree of complexation of Cu(II) and Fe(II) by flavones and noted, that their ability to chelate copper ions was higher. Kumamoto *et al.* [33] determined acid dissociation constants of free and metal complexed four catechins. Binding with Cu(II) and Fe(II) caused much greater decrease in acid dissociation constant of catechins than complexation with Zn(II). Zinc ions were found to be precipitated by tannins to a much weaker extent than copper ions [34].

A number of studies on chelation of metal ions, especially copper and iron, by isolated phenolic compounds *i.e.*, phenolic acids, flavonoids, anthocyanins have established that suitably oriented functional groups in the structure of ligand are essential for formation of metal ion–phenolic compound complexes [32,35–37]. Phenolic compounds with a single OH group on the aromatic ring do not bind copper and iron ions [36,38]. The presence of a catechol group (*o*-dihydroxyphenyl) or galloyl group (trihydroxyphenyl) is essential to complex metal ions. At the same time, iron ions preferentially bind to three phenol groups of the gallate moiety, and *o*-dihydroxyphenyl groups play a crucial role in complex formation with copper [35,38,39]. When the chemical structure of tannins is considered, it could be presumed that the tannin fraction from walnuts, containing mainly hydrolysable tannins, would better chelate Fe(II) ions, than tannin fractions from almonds and hazelnuts, rich in proanthocyanidins. In turn Cu(II) should be much better bound by the numerous catechol groups of condensed tannins of almonds and hazelnuts. Such simple relationships were not noted in the case of tannin fractions tested, probably due to much more complex structure of their constituents. For example it is known, that glycosylation of OH group of phenol prevents metal from binding [40]. Almonds and hazelnuts proanthocyanidins contain (epi)catechin glycosides as subunits [24], what can diminish their ability to complex Cu(II). On the other hand, the presence of methoxyl and hydroxyl groups in the *ortho* position increases Cu(II) chelation capacity [37]. It was also noted that structure does not influence complexing of iron ions [38].

## 3. Experimental Section

### 3.1. Materials

The raw seeds of hazelnuts, walnuts and almonds with their skins on were acquired at local market in Olsztyn. Poland.

### 3.2. Chemicals and Reagents

Sephadex LH-20, vanillin, tannic acid, gallic acid, (+)-catechin, BSA (bovine serum albumin), hexamine (hexamethylenetetramine), TMM (tetramethylmurexide ammonium salt), ferrozine (3-(2-pyridyl)-5,6-diphenyl-1,2,4-triazine-4’,4”-disulfonic acid) were purchased from Sigma-Aldrich Co. Ltd. (Pozna, Poland). Procyanidin B_2_ was obtained from Extrasynthese S.A. (Genay Cedex, France). HPLC solvents were acquired from Merck Co. (Darmstadt, Germany). Potassium chloride (KCl), ferrous chloride tetrahydrate (FeCl_2_ × 4H_2_O), cupric sulfate pentahydrate (CuSO_4_ × 5H_2_O), zinc chloride (ZnCl_2_), acetone, ethanol and other reagents, all analytical grade, were obtained from P.O.Ch. Co. (Gliwice, Poland).

### 3.3. Isolation of Tannin Fractions

Ground and defatted with hexane hazelnuts, walnuts and almonds were extracted with 80% acetone (v/v) according to procedure of Amarowicz *et al.* [41]. Lyophilised extracts (2 g) were dissolved in 20 mL of ethanol and applied into a column (5 × 40 cm) packed with Sephadex LH-20 gel. At first low molecular weight phenolic compounds were eluted with ethanol (1 L). Part of lower molecular hydrolysable tannins as well as procyanidins, including most momoners and some dimmers and trimers could have been coeluted in this step. Then 600 mL of acetone:water (1:1, v/v) was used to elute tannins [42]. Acetone was removed using rotary evaporator, and aqueous residue was lyophilised.

### 3.4. Content of Condensed Tannins

The content of condensed tannins in fractions was determined according to a modified vanillin assay [43]. To 1 mL of fractions dissolved in methanol (at a concentration of 1 mg/mL), 5 mL of vanillin/HCl reagent [0.5 g vanillin in 4% hydrochloric acid in methanol (v/v)] was added. Absorbance at 500 nm using Beckman DU 7500 diode array spectrophotometer (Beckman Instruments Inc., USA) was recorded after 20 min of samples standing in darkness. Parallel control samples (without vanillin) were prepared. Results were calculated using the standard curve for (+)-catechin (0.2–0.8 mg/mL, *r*^2^ = 0.997) and expressed as mg catechin equivalents (CE) per gram of tannin fraction.

### 3.5. Protein Precipitation Capacity

The bovine serum albumin (BSA) precipitation ability of samples was examined using method developed by Hagerman and Butler [44]. Briefly, to 2 mL of BSA (concentration of 1 mg/mL in 0.2 M acetic buffer, pH 5.0 with 0.17 M NaCl) 1 mL of tannin fractions in water (1 mg/mL) was added. Samples were allowed to stand for 15 min at room temperature and then were centrifuged at 5,000 g for 15 min. The supernatant was removed, and pellet was dissolved in 4 mL of water solution of 1% SDS and 5% triethanolamine. Then 1 mL of 0.01 M FeCl_3_ in 0.01 M HCl was added. The samples were left for 30 min at room temperature and the absorbance at 510 nm was measured. Results were calculated using the standard curve for tannic acid (0.2–1.0 mg/mL, *r^2^* = 0.996) and expressed as mg tannic acid equivalents (TAE) per gram of tannin fraction.

### 3.6. SE-HPLC Method

A SE-HPLC method was employed to examine the molecular weight distribution of nut tannin fractions obtained from Sephadex LH-20 separation. The samples were dissolved in 45% acetonitrile (v/v) with 0.1% TFA (v/v) at concentration 2 mg/mL and analysed using a Shimadzu HPLC system (Shimadzu Co., Japan) consisting of LC-10AD_Vp_ pumps, SCL-10A_Vp_ system controller and UV-VIS SPD-M10A_Vp_ photo-diode array detector. The TSK-GEL^®^ G2000SW_XL_ column (7.86 × 300 mm, 5 μm, Tosoh Co., Japan) was employed. The mobile phase of 45% acetonitrile with 0.1% TFA was delivered at a rate of 0.2 mL/min. The detection was monitored at 280 nm and the injection volume was 20 μL. Gallic acid, procyanidin B_2_ and tannic acid with molecular weights 170, 578 and 1701, respectively, were used as standards.

### 3.7. Chelation Capacity

In order to determine Cu(II) and Zn(II) chelation capacity, tannin fractions were dissolved in 0.01 M hexamine/HCl buffer with addition of 0.01 M KCl, pH 5.0 [30]. The samples were diluted in the range 0.2–1.0 mg/mL or 1–5 mg/mL to determine bound Cu(II) and Zn(II), respectively. CuSO_4_ × 5H_2_O or ZnCl_2_ were dissolved in the same buffer at concentration of 0.25 mM and 0.8 mM, respectively. Tannin solution (1 mL) was mixed with 1 mL of salt solution, then 0.1 mL of tetramethylmurexide at concentration of 1 mM was added. Absorbance was recorded at 482 nm [Cu(II)] or 462 nm [Zn(II)] and 530 nm [Cu(II) and Zn(II)] and the ratio of A_482_/A_530_ [Cu(II)] or A_462_/A_530_ [Zn(II)] was calculated. Control samples were prepared in the same way: redistilled water was added instead of TMM reagents. A standard curve of absorbance ratio *vs.* metal ions concentration was prepared; Cu(II) in the range of 0.025–0.125 mM and Zn(II) from 0.2 to 2.0 mM. The percentage of bound metal ions was calculated.

Fe(II) chelation activity of tannin fractions from hazelnuts, walnuts and almonds was examined using ferrozine [45]. Tannin fractions were dissolved in water at a concentration in the range of 0.2–1.0 mg/mL. Solutions (2.5 mL) were mixed with 0.25 mL of 0.4 mM FeCl_2_ × 4H_2_O, then 0.5 mL of 5 mM ferrozine was added. Then reaction mixture was left for 10 min at room temperature and absorbance at 562 nm was measured. Control samples were prepared in the same way, but water was added instead of ferrozine solution. The percentage of Fe(II) bound was calculated.

## 4. Conclusions

This study revealed that tannin fractions obtained from walnuts, hazelnuts and almonds seeds by Sephadex LH-20 column chromatography were able to chelate copper and iron ions, while zinc ions were only bound significantly by almond and hazelnut tannins. Among the metal ions tested, the effectiveness of chelation by the constituents of nut tannin fractions decreased in the order Cu(II) > Fe(II) > Zn(II). The exception was the case of tannin from almonds, which chelated more zinc than iron ions. The chelating capacity of individual tannin fractions analysed varies for different complexed metals and it cannot be pointed out which fraction is the best chelator of metal ions. No relationship was found between molecular weight or type of tannins present in isolated nut fractions and their ability to chelate Cu(II), Fe(II) and Zn(II). Tannin fraction from walnuts containing mainly hydrolysable tannins hardly complexed Zn(II), whereas its Cu(II) chelating capacity was much higher than the fractions isolated from almonds and hazelnuts, which comprise mainly proanthocyanidins. Oligomers of hazelnuts tannins which possess higher molecular weights than condensed tannins of almonds fraction, complexed Cu(II) stronger, but Fe(II) and Zn(II) less effectively.

## Figures and Tables

**Table 1.** Condensed tannins content and protein precipitation capacity of nuts tannin fractions.

**Nuts tannin fraction**	**Condensed tannins (mg CE^a^/g)**	**Protein precipitation capacity (mg TAE^b^/g)**
Walnuts	147 ± 4	873 ± 12
Hazelnuts	1261 ± 12	940 ± 24
Almonds	776 ± 8	69 ± 9

Mean ± standard deviation (*n* = 3). Results are expressed as equivalents of standard per g of tannin fraction.

a Catechin equivalents.

b Tannic acid equivalents.

**Table 2.** Ability of tannin fractions of nuts to chelate copper, iron and zinc ions.

**Nuts tannin fraction**	**μmol metal ions chelated by 0.5 mg of tannin fraction**		
	**Fe(II)^a^**	**Cu(II)^b^**	**Zn(II)^b^**
Walnuts	2.7 ± 0.08	25.0 ± 0.90	0.1 ± 0.01
Hazelnuts	1.8 ± 0.11	22.5 ± 1.03	1.5 ± 0.08
Almonds	3.2 ± 0.14	20.5 ± 0.89	11.4 ± 0.57

Mean ± standard deviation (*n* = 3).

a Determined by ferrozine assay.

b Determined by tetramethylmurexide assay.
